# No evidence for spectral jamming avoidance in echolocation behavior of foraging pipistrelle bats

**DOI:** 10.1038/srep30978

**Published:** 2016-08-09

**Authors:** Simone Götze, Jens C. Koblitz, Annette Denzinger, Hans-Ulrich Schnitzler

**Affiliations:** 1 University of Tuebingen, Department Animal Physiology, Institute for Neurobiology, Auf der Morgenstelle 28, 72076 Tuebingen, Germany; 2 BioAcoustics Network, Neuss, Germany

## Abstract

Frequency shifts in signals of bats flying near conspecifics have been interpreted as a spectral jamming avoidance response (JAR). However, several prerequisites supporting a JAR hypothesis have not been controlled for in previous studies. We recorded flight and echolocation behavior of foraging *Pipistrellus pipistrellus* while flying alone and with a conspecific and tested whether frequency changes were due to a spectral JAR with an increased frequency difference, or whether changes could be explained by other reactions. *P. pipistrellus* reacted to conspecifics with a reduction of sound duration and often also pulse interval, accompanied by an increase in terminal frequency. This reaction is typical of behavioral situations where targets of interest have captured the bat’s attention and initiated a more detailed exploration. All observed frequency changes were predicted by the attention reaction alone, and do not support the JAR hypothesis of increased frequency separation. Reaction distances of 1–11 m suggest that the attention response may be elicited either by detection of the conspecific by short range active echolocation or by long range passive acoustic detection of echolocation calls.

Both technical and biological active sensing systems such as radar, sonar, and echolocation in bats and cetaceans are based upon the same principle; a sender transmits signals and a receiver analyzes the returning echoes to detect, localize, and classify targets of interest. Echolocating bats emit high frequency signals and decode information in the returning echoes to navigate in space and to find their food. The evaluation of returning echoes, however, may be hindered if signals from other sources interfere with the pulse echo train that encodes the position and nature of the target of interest. For technical sensing applications, such jamming signals can be purposeful; for instance, in radar and sonar anti-warfare systems strong signals are used to block the receiver of the opponent’s active sensing device. In biosonar systems inadvertent signals from nearby conspecifics can also have a jamming effect. These species-specific signals are rather similar in their frequency structure, such that masking effects might be possible.

Countermeasures to avoid jamming could include varying either frequency or timing of the emitted signal. In order for jamming avoidance to be possible in the frequency domain (spectral jamming avoidance), the relevant frequencies must be sufficiently different such that processing occurs in distinct non-interfering frequency channels of the receiver. In the time domain, signals have to be separated sufficiently in time such that a clear attribution of an emitted pulse to the echoes of this pulse is possible (temporal jamming avoidance).

Spectral variations found in signals and temporal variations in the emission pattern of bats flying in the same area have each been interpreted as jamming avoidance strategies. Spectral jamming avoidance has been discussed for *Rhinopoma hardwickei*^1^, *Euderma maculatum, Eptesicus fuscus, Lasiurus borealis, Lasiurus cinereus*^2^, *Eptesicus fuscus*^3^,^4^, *Tadarida teniotis*^5^, *Tadarida brasiliensis*^6^, *Balantiopteryx plicata*^7^, and for 3 species of pipistrelle bats^8^,^9^,^10^. An increase of the terminal frequency in echolocation signals of flying bats in response to playback signals was also considered evidence of a spectral jamming avoidance response (JAR) in *T. brasiliensis*^11^, and in *Pipistrellus abramus*^12^. Bates *et al.*^13^ confronted stationary *E. fuscus* with a constant frequency jamming tone while they performed a two-alternative forced-choice detection task. All bats shifted the terminal frequency of their signals away from the jamming signal, as long as the jamming frequency was sufficiently close to their preferred frequency (within 2–3 kHz), whereas signal duration did not change.

Possible temporal jamming avoidance has been reported from interactions in flying bats for *E. maculatum, E. fuscus, L. borealis, and L. cinereus*^2^. In addition, long pulse intervals in *E. fuscus* (>200 ms) when flying with conspecifics in a flight room^14^, mutual suppression of pulse emissions in groups of *T. brasiliensis* positioned in a small flight cage^15^, and more frequent signal emissions in the silent period between jamming signals in *P. abramus*^12^ have been interpreted as temporal jamming avoidance reactions. The suppression of pulse emission after regularly repeated acoustic stimuli in stationary *T. brasiliensis* may also be evidence of temporal jamming avoidance by antiphonal pulse emission^16^.

Previous research has indicated that not all bat species react to conspecifics with a jamming avoidance response. *Taphozous perforatus*^5^ and *Craseonycteris thonglongyai*^17^ did not shift the frequency of their echolocation signals in the presence of conspecifics. In a recent paper, Cvikel *et al.*^18^ present data from foraging *Rhinopoma microphyllum* encountering nearby conspecifics. They recorded the echolocation signals of both bats with an on-board miniature microphone and found changes in echolocation behavior, including frequency shifts, which they interpret as bats responding to other bats “as though they were nearby objects rather than avoiding being jammed by them”.

The current accepted paradigm in the field of echolocation in bats is that echolocating bats react to interfering signals from conspecifics with spectral jamming avoidance. We doubted the evidence that support this conclusion, as the results often left important questions unanswered. Most previous studies have only shown changes in terminal frequencies of interacting bats, but not an increased frequency difference, which is a relevant prerequisite to support spectral jamming avoidance. Further, the spatial relation between the recorded bats (which has a strong influence on echolocation behavior) was mostly unknown. Therefore we hypothesize-similar to the conclusion in the recent publication of Cvikel *et al.*^18^ - that the reported changes in these studies are not evidence of JAR, but rather have the function of exploring near-by conspecifics.

All bats vary their echolocation behavior according to the performed tasks. Bats either show approach behavior when they close in on prey, land, or pass an obstacle (summarized in^19^), or they react to background targets such as vegetation and the ground (summarized in^20^). In both behavioral situations, distance to the target of interest is the most relevant factor determining echolocation behavior. The patterning, frequency, harmonic structure, bandwidth, duration, pulse interval, and source level (SL) of the echolocation signals are systematically varied depending on the distance to the prey or background^3^,^19^,^20^,^21^,^22^,^23^,^24^,^25^,^26^,^27^,^28^,^29^. In general, duration, pulse interval, and SL of the search signals are reduced and signal bandwidth is increased with decreasing target distance.

To test the hypothesis that reactions to conspecifics do not indicate jamming avoidance but can instead be explained as explorative behavior directed towards the other bat we reconstructed flight paths of pipistrelle bats (*P. pipistrellus*) and compared their echolocation behavior while flying alone and with a conspecific, considering their inter-individual distance. We analyzed whether changes in terminal frequency support the JAR hypothesis or whether they have another function. According to the JAR hypothesis, signal frequencies should be shifted apart in reacting bats. Alternatively, according to our hypothesis, the observed changes should reflect a switch to another echolocation task such as the exploration of a near-by conspecific, which has captured the attention of the reacting bat.

The signal repertoire of many bat species including pipistrelle bats is species-specific. This specificity is mainly reflected in the peak^30^ and terminal frequency of the search signals. Within each species, the parameters of sound duration and terminal frequency are negatively correlated as already described for *P. pipistrellus*^25^,^27^. Similar correlations were also found in other vespertilionid bats^3^,^31^,^32^,^33^,^34^,^35^. Within species, individual bats differ somewhat in their signal parameters, which is particularly evident when comparing the terminal frequency of their signals^2^,^8^,^9^,^13^,^23^,^27^,^36^,^37^,^38^. Here, we determined the species-specific call repertoire of *P. pipistrellus* and of individual bats, and tested if it is the same in bats flying alone and together.

## Material and Methods

### Species and study sites

Echolocation and flight behavior of foraging common pipistrelle bats (*Pipistrellus pipistrellus*) were observed from July to October 2010 in Bebenhausen and Ofterdingen, Baden-Württemberg, south-western Germany. The echolocation signals of pipistrelles foraging near street lights were recorded for four hours starting at dusk in 12 nights, producing a total of 48 hours of recording time. The recording sites were positioned at the periphery of residential areas in the vicinity to orchards and at distances of 10–20 m to houses. Houses and trees created edge space foraging habitats which were connected to open space areas above meadows and streets. The number of recorded bats remains unknown as we conducted this study with free flying bats in the field. Several recordings revealed echolocation signals of up to six individuals at the same time. We therefore assume that at least six individuals foraged at the recording sites. We analyzed 20 pairs of bats flying at the same time.

### Sound recordings

We used a horizontally oriented T-shaped and planar microphone array consisting of four microphones (Knowles, Model SPM0404UD5). Three microphones were positioned in a line with interspaces of one meter and a fourth was fixed at a right angle to the central microphone at a distance of one meter. The array was adjusted to heights of 1.2–1.6 m above ground and positioned near streetlights with foraging bats.

Each of the sound recordings of the four microphones was amplified, digitized with a sampling rate of 250 kHz with an A/D-converter (National Instruments, Texas, Type USB-6251), and stored as a wav file using the custom made software ‘Battery’.

### Data analysis

Recorded signals were visualized as color spectrograms (FFT 512, Blackman window, dynamic range of 90 dB) using custom-made software (Selena, University of Tübingen, Germany). The spectrograms were plotted with a temporal resolution of 0.06 ms and a spectral resolution of 117.5 Hz due to auto-padding and time interpolation. The beginning and end of signals were measured in the spectrograms using the automatically applied criterion of −20 dB below highest amplitude. In a few signals with poorer signal-to-noise ratios, the criterion was reduced to −15 or −10 dB. Signal parameters including duration, pulse interval, peak frequency, and terminal frequency were measured.

### The species-specific transmission channel of the terminal frequency

**Figure 1:**
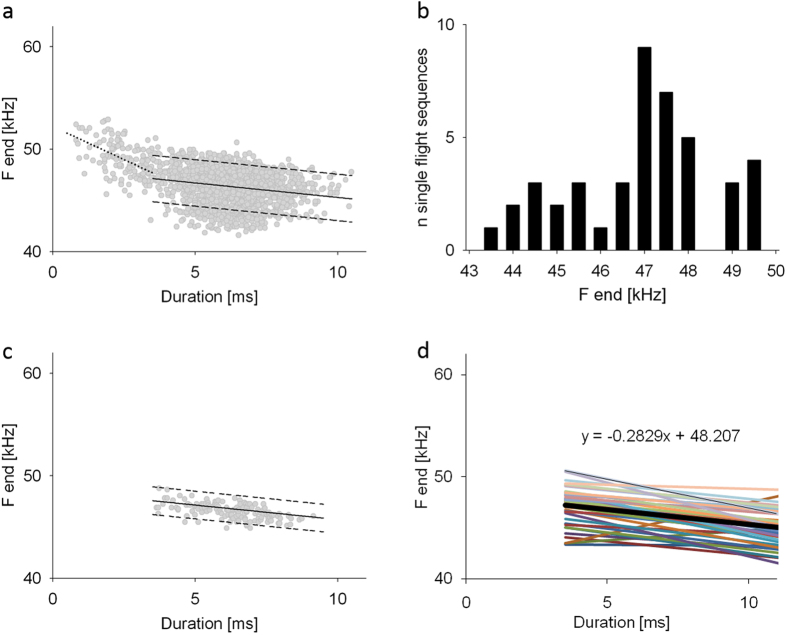
(**a**) Search (≥3.5 ms) and approach calls (<3.5 ms) from 43 sound sequences (2147 calls) of single flying *P. pipistrellus* with linear regressions (solid and dotted line, respectively). The dashed lines indicate the twofold standard deviation and limit the species-specific transmission channel for search signals. (**b**) Distribution of individual terminal frequencies (measured as average frequencies of calls with durations of 5–6 ms) of single flying individuals of *P. pipistrellus*. (**c**) Individual transmission channel of the terminal frequency of a single flying *P.pipistrellus* in search flight. (**d**) Linear regressions for the search calls of the analyzed 43 single flight sequences. Thicker black line indicates mean of all individual regressions.

Echolocation calls of *P. pipistrellus* show a negative correlation of call duration and terminal frequency. Signals with longer durations have lower terminal frequencies than shorter calls. Visualization of the relation between these two parameters reveal that all signals are concentrated in a rather small frequency band which we will call the transmission channel of the terminal frequency (Fig. 1). Here we use the transmission channel for the characterization of the species-specific signal repertoire. Baumann^34^ was the first who used this method to describe the signal repertoires of *Eptesicus serotinus* and *Eptesicus nilssoni.*

For the determination of the transmission channel we correlated terminal frequency with call duration, performed regression analysis, and calculated the residuals of all data points. The slope of the resulting regression line indicates how terminal frequency changes with sound duration. The width of the transmission channel is given by twice the standard deviation of the residuals and channel position is indicated by the location of the regression line in the frequency axis. With this approach we determined the species-specific transmission channel by using all search signals from all analyzed sound sequences recorded in single flights (Fig. 1A). We also determined individual transmission channels using sequences of successive search signals from single bats (Fig. 1C).

For the determination of transmission channels, we discriminated between search and approach signals. Calls with durations of 3.5 ms or greater were counted as search calls, whereas those shorter than 3.5 ms were considered approach signals. Pulse intervals > 15 ms excluded all buzz signals^27^. For species-specific transmission channel calculations, we analyzed echolocation calls of single flying *P.pipistrellus*. Recordings were conducted at different recording sites and at different evenings to minimize replications of few individual bats.

The transmission channel for the terminal frequency of an individual *P. pipistrellus* is described by its slope, width, and position in the frequency range. For the description of the terminal frequency of individual bats, we used the average frequency value of a hypothetical 5.5 ms long signal, which we calculated as the mean terminal frequency of all signals with duration of 5–6 ms (Fig. 1B).

We then constructed an average individual transmission channel using the average slope and width of all measured individual transmission channels (Fig. 1C). If this channel is positioned at the 5.5 ms frequency value of an individual it can be used as a prediction for the duration-frequency relation of further search signals. We determined the 5.5 ms value from sounds of single flight sequences of individual bats, adjusted the channel to this frequency, and tested whether the signals emitted when flying together with another bat were also within this predicted individual transmission channel. A channel width of two standard deviations accounts for 95% of the data.

### 3D-flight path reconstruction

Calls of *P. pipistrellus* flying above the T-shaped array reached the microphones at different times due to the different distances between the bat and the microphones. These differences in arrival time encode the spatial position of the bat in the moment of call emission relative to the array. Time-Of-Arrival-Differences (TOAD) were determined by measuring the time lag at which the cross-correlation function between the signal of the reference microphone and the signal of each of the other three microphones reached the maximum value^39^. The three TOADs allowed us to determine the three-dimensional positions of bats above the array. The positions from consecutive calls of individual bats were then assembled to determine individual flight paths.

### Differentiation of single flights and flights with conspecific

Sound sequences from a single bat produced a single reconstructed flight path whereas sequences with signals from two bats led to two separate flight paths. Thus, it was possible to attribute each signal of a sound sequence to its sender. In flights with two bats, we measured inter-individual distances. The criterion for a ‘flight with conspecific’ was set to an inter-individual distance of ≤10 m. Sequences of two pipistrelles with inter-individual distances of >10 m were classified as ‘single flights’. For each ‘flight with conspecific’ (n = 20) we distinguished between two scenarios, encounter and pursuit. During encounters, the two bats approached each other more or less head-on and faced their conspecific at an angle of 0–90° from their actual flight direction. In pursuits, one bat flew behind its conspecific and both flew more or less in the same direction. The criterion for pursuit was defined by the angle between the flight directions of pursuer and pursued bat being less than 90°. For the description of the interaction behavior, we identified the bat that was already circling around the light source as “resident” and the joining conspecific as “intruder”.

### Identification of reactions to nearby conspecifics

The criterion for the beginning of a reaction to a nearby conspecific was a reduction of pulse duration and/or pulse interval by at least 10% in relation to the mean value of the preceding five calls. Changes in terminal frequency were not used to prevent a circular argument. Reaction strength varied over a wide range. At weakest reactions, the regular pattern of search signals was interrupted by only one signal with reduced duration and interval, whereas strong reactions were characterized by reduced pulse durations below 3 ms for longer time spans, and by the emission of groups of signals with shorter pulse intervals instead of the single signals with intervals around 90 ms that are typically emitted.

### Statistics

For all bats reacting in flight to a conspecific, three parameters (call duration, pulse interval and terminal frequency) of the last five search calls before reaction were compared with the parameters of the following five calls after the beginning of the reaction by running a non-parametric Wilcoxon signed-rank test per bat and an ANOVA (using JMP 11.2.0) for the two groups of calls (prior to reaction and after beginning of reaction). The significance level of the comparison between the parameters call duration and pulse interval before and after beginning of reaction served as a classification of reaction strength as strong (p < 0.01), medium (p < 0.05), or weak reactions (0.05 < p < 0.1). Reactions without significant differences in their parameters were also classified as weak reactions if call duration and/or pulse interval were reduced by at least 10% in relation to the mean value of the preceding five calls.

We calculated a Spearman rank correlation coefficient to test for correlations between shifts in terminal frequency after beginning of reactions and absolute terminal frequency differences between individual bats, which were determined from single flight calls with durations of 5–6 ms.

The regression between call durations and terminal frequencies was calculated and compared for both search and approach calls by performing an ANCOVA (using JMP 11.2.0).

## Results

### Echolocation behavior and transmission channels of single flying bats

At both recording sites, pipistrelle bats foraged near street lamps with similar flight and echolocation behavior. In most cases, single bats circled around or near the street lamps at altitudes between three and ten meters. Now and then, an additional bat (intruder) would pass by and interact with the bat already circling the light source (resident). Bats foraging alone showed typical search behavior, which was occasionally interrupted by typical approach behavior indicating an insect pursuit^27^. In search flights echolocation signals with durations of up to 9 ms were emitted at pulse intervals around 90 ms, most likely in the rhythm of wingbeat. Interspersed pulse intervals around 180 ms indicated wingbeats without sound emission.

The species-specific transmission channel of single flying *P. pipistrellus* was calculated from 43 sound sequences with 2147 echolocation calls (Fig. 1A). Slope and position of the species-specific transmission channel of search calls were described by the equation y = −0.2829 × +48.123 and differed significantly (ANCOVA: F(3,2147) = 26.9, p < 0.0001, Cohen’s d = 0.46) from the regression of the approach calls (y = −1.2911 × +52.209). Individual transmission channels were also calculated for each of the analyzed 43 sequences (Fig. 1C). The mean slope of all individual regressions was the same (y = −0.2829 × +48.207, Fig. 1D) as the corresponding value of the species-specific regression calculated from all calls of the 43 sequences (Fig. 1A). With 4.5 kHz (±1.133 * 2, twofold s.d. of the residuals), the width of the species-specific transmission channel was distinctly larger than the mean width of 2.7 kHz (±0.6744 * 2, twofold s.d. of the residuals) for all individual transmission channels. The calculated average species-specific terminal frequency of signals with duration of 5.5 ms was 46.5 kHz. Individual frequencies related to signal durations of 5.5 ms were distributed over a range from 43.5–49.1 kHz, indicating that recordings were taken from multiple individuals. However, pseudoreplication cannot be completely excluded, as some sequences had similar terminal frequencies (Fig. 1B) and regression lines (Fig. 1D).

The measured emission frequencies contained Doppler shifts resulting from a subject’s horizontal flight speed vector towards the microphone, and from the elevation angle between subject and microphone. We are aware that our frequency data still contain Doppler shifts so that the measured frequency could deviate under the most unfavorable conditions at a maximal flight speed vector of 6 m/s by up to 0.6 kHz from the emission frequency. However, in most measurements the Doppler error was smaller, as most recordings were taken from subjects flying directly above the array. Additionally, as the bat´s movements produced positive as well as negative Doppler shifts, it is unlikely that our averaged data contain any directional biases.

### Echolocation behavior and transmission channels of interacting bats

Mostly resident and intruder did not react to each other in neither their flight nor echolocation behavior if the distance between them was greater than 10 m. In encounters, where the bats flew more or less towards each other, both bats often reacted distinctly in their echolocation behavior (and occasionally in their flight behavior) whereas in pursuits, where one bat followed the other bat, often only the pursuer showed a distinct reaction. Here we present data of eleven encounters and nine pursuits and compare the echolocation behavior of the bat’s single flights with their behavior recorded during flights with the conspecific.

**Figure 2:**
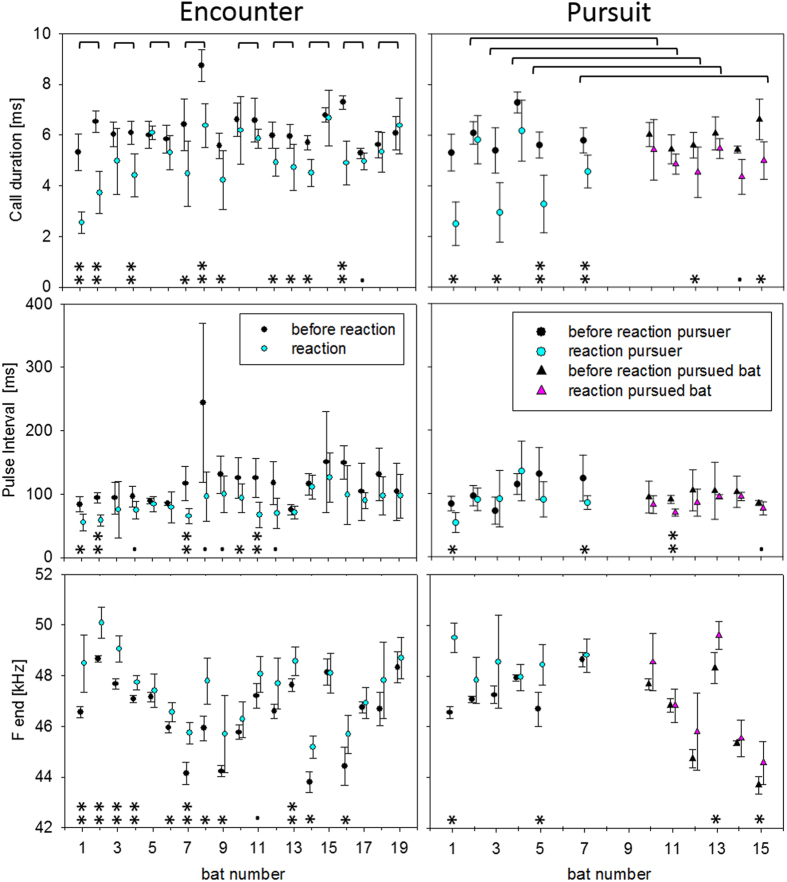
Reactions to conspecifics as indicated by changes in call duration, pulse interval and terminal frequency. For all bats reacting in flight to a conspecific the three call parameters (call duration, pulse interval and terminal frequency; mean ± s.d.) of the last five search calls before reaction (black) were compared with the parameters of the following five calls after beginning of the reaction (blue and pink). Data for encounters (bats fly towards each other) and pursuits (one bat flies behind the other) are presented separately. Pursuits are additionally separated into reactions of the pursuer (blue) and the pursued bat (pink). The inter-acting bat pairs are marked by parentheses above the data. The significance level of the comparison between parameters before and after beginning of reaction is indicated below (**p < 0.01; *p < 0.05;. = Trend (0.05 < p < 0.1)).

We identified reactions of both bats in nine encounters and one additional encounter where only one bat reacted. We also found six pursuit situations where both bats fulfilled the criterion conditions for the beginning of a reaction and one more where only one bat reacted (Tables 1 and 2, Fig. 2). The reaction of the pursuer in sequence 14 (Bat 1, Table 1) is not shown in Fig. 2 due to an insufficient number of calls prior to reaction (n < 5 calls). In one more encounter situation and in two more pursuit conditions both bats did not react to each other at inter-individual distances below 10 m, even at a minimal distance of 3.5 m.

**Figure 3:**
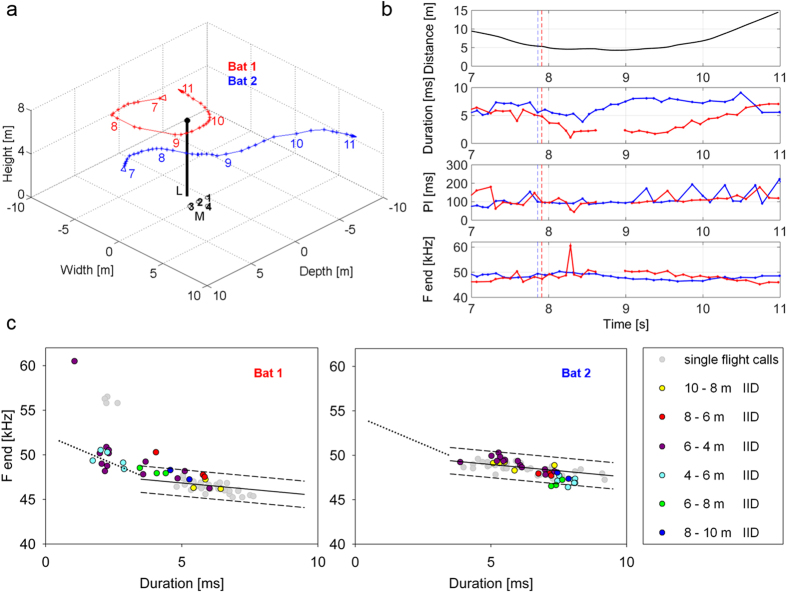
(**a**) 3D plot of the flight paths where Bat 1 pursued Bat 2. Open arrows indicate the start and filled arrows the end of the flight path. Time (in seconds) is marked by numbers. L = street light, M = microphone array. (**b**) Inter-individual distance and call parameters of Bat 1 (red) and Bat 2 (blue). The dotted vertical lines mark the beginning of the bats’ reaction in echolocation behavior. (**c**) Correlation of terminal frequency with call duration of Bat 1 and Bat 2 from signals emitted in single flight (grey dots) and in flight with the conspecific (colored dots). The single flight signals were used to position the individual transmission channels (between dashed lines) at the 5.5 ms frequency values. The color of the dots indicates groups of inter-individual distances (IID). The pursuing Bat 1 reacted at a distance of 5.3 m with a distinct reduction of pulse duration and interval which continued up to t = 10.5 s, after both bats had turned away from one another. At about the same distance, the pursued Bat 2 reacted with a minor reduction of pulse duration which ended at t = 8.5 s, when it was ahead of Bat 1. During the flight with the conspecific, the frequency of nearly all signals of Bat 1 and Bat 2 were within the transmission channels. The few signals outside the transmission channels even reduced the frequency difference between Bat 1 and Bat 2. Bat 1 also emitted one short, high frequency call while reacting to Bat 2.

**Figure 4:**
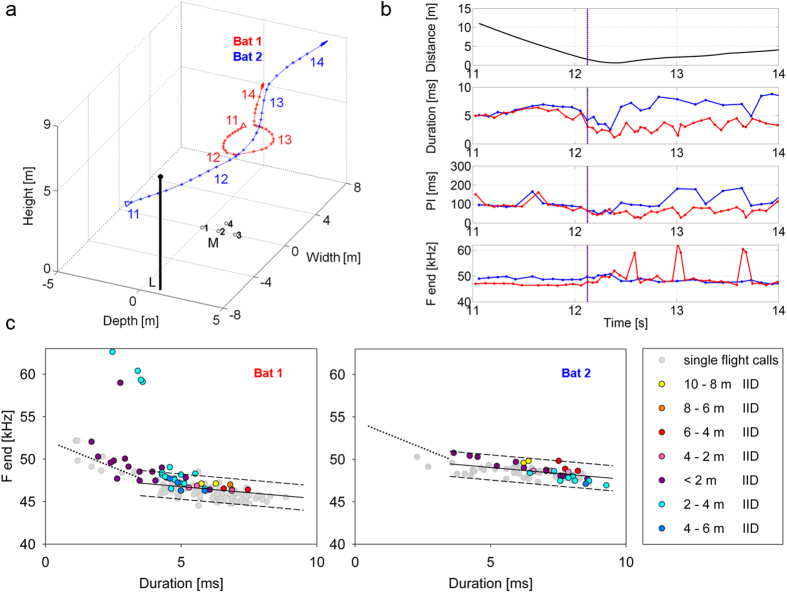
(**a**) 3D plot of the flight paths. Open arrows indicate the start and filled arrows the end of the flight path. Time (in seconds) is marked by numbers. L = street light, M = microphone array. (**b**) Inter-individual distance and call parameters of Bat 1 and Bat 2. The dotted vertical lines mark the beginning of the bats’ reaction in echolocation behavior. (**c**) Correlation of terminal frequency with call duration of Bat 1 and Bat 2 from signals emitted in single flight (grey dots) and in flight with the conspecific (colored dots). The single flight signals were used to position the individual transmission channels (between dashed lines) at the 5.5 ms frequency values. The color of the dots indicates groups of inter-individual distances (IID). During the encounter the two bats reacted to each other at a rather short distance (1.5 m) by a distinct reduction in sound duration and pulse interval. During the pursuit the leading Bat 2 switched back to normal search behavior with long sound durations and pulse intervals whereas the pursuer (Bat 1) continued its reaction as indicated by signals with short durations and pulse intervals. During the flight with the conspecific, the frequency of nearly all signals of Bat 1 and of all signals of Bat 2 were within the predicted individual transmission channel. Bat 1 also emitted a few short and high frequency calls while reacting to Bat 2.

**Figure 5:**
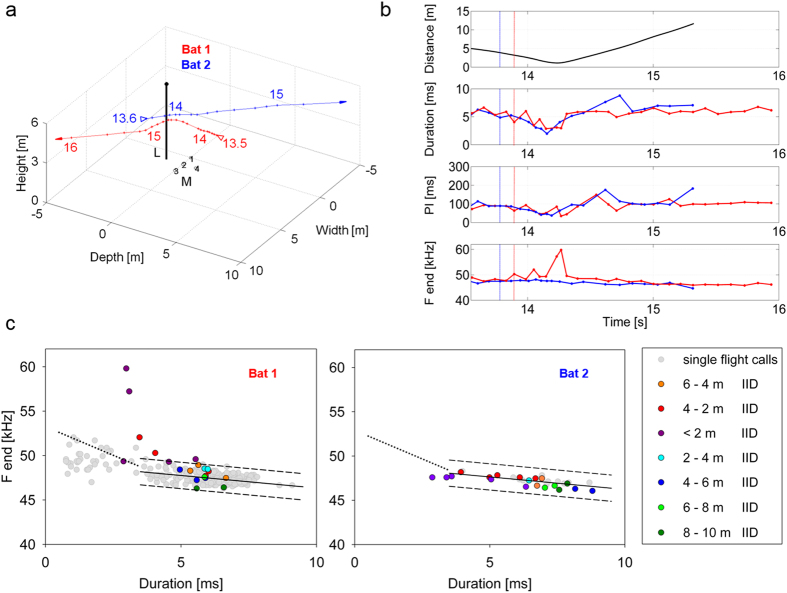
(**a**) 3D plot of the flight paths of two *P.pipistrellus*. Open arrows indicate the start and filled arrows the end of the flight path. Time (in seconds) is marked by numbers. L = street light, M = microphone array. (**b**) Inter-individual distance and call parameters of Bat 1 (red) and Bat 2 (blue). The dotted vertical lines mark the beginning of the bats’ reaction in echolocation behavior. (**c**) Correlation of terminal frequency with call duration of Bat 1 and Bat 2 from signals emitted in single flight (grey dots) and in flight with the conspecific (colored dots). The single flight signals were used to position the individual transmission channels (between dashed lines) at the 5.5 ms frequency values. The color of the dots indicates groups of inter-individual distances (IID). Both bats reacted at about the same distance (Bat 1 at 3.2 m and Bat 2 at 3.9 m) with a reduction of sound duration and pulse interval. Bat 1 also emitted a few short and high frequency calls while reacting to Bat 2. After the encounter both bats went back to normal search behavior with call durations > 5 ms. During the flight with the conspecific the frequency of nearly all signals of Bat 1 and of all signals of Bat 2 were within the transmission channel.

The reaction patterns differed strongly and ranged from the emission of one or a few slightly shorter single signals still in the rhythm of wingbeat (e.g., weak reaction of Bat 2 (blue) at t = 7.9 s on, Fig. 3B) to longer-duration signals in series, arranged in groups of two or three with pulse intervals distinctly below the 90 ms of the wingbeat cycle (e.g., strong reaction of Bat 1 (red) at t = 12.1 s on, Fig. 4B). The reaction distances varied over a wide range (between 1–11 m; Tables 1 and 2; Figs 3, 4, 5). The mean reaction distance was 4.6 ± 2.5 m in pursuits and 5.5 ± 2.8 m (mean ± s.d.) in encounters.

In all reacting bats the change in duration and/or pulse interval by at least 10% in relation to the mean value of the preceding five calls was accompanied by an increase of the signal parameter terminal frequency. In 29 of the 32 observed reactions the frequency was at least 122 Hz higher in the reaction calls and only in three reactions it was about the same (Fig. 2).

During reactions of 15 pairs of bats, the differences between the terminal frequencies of the involved individuals decreased in seven of nine pairs in encounters and in four of six pairs in pursuits (73.3%). The frequency changes were independent of the absolute difference between the individual frequencies of the reacting bats (Spearman’s ρ, R = −0.03, N = 31, p = 0.88; Supplemental Material Fig. S1).

**Figure 6:**
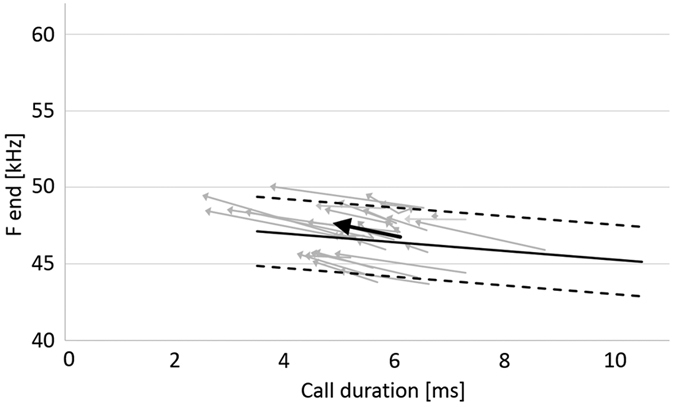
The data presented in Fig. 2are used to calculate the individual reaction vectors. Positions and slopes of the reaction vectors are predicted by the species-specific transmission channel. Reacting bats reduced call duration on average by 1.27 ± 0.09 ms and increased terminal frequency by 0.95 ± 0.12 kHz (mean ± s.d.). Call duration and terminal frequency before and after beginning of reaction differed significantly (ANOVA, p < 0.0001).

To describe the relation between the reduction in call duration and the increase in terminal frequency we calculated a vector for all individual reactions and for their mean values before and after beginning of a reaction and compared them with the species-specific transmission channel (Fig. 6). A significant increase of terminal frequency by 0.95 ± 0.12 kHz (mean ± s.d.; ANOVA, F(1,314) = 25.68, p < 0.0001) was found at a significant reduction of call duration by 1.27 ± 0.09 ms (mean ± s.d.; ANOVA, F(1,312) = 96.95, p < 0.0001). Position and slopes of the individual reaction vectors and of the mean vector are predicted by the species-specific transmission channel (Fig. 6).

In individual reactions the reduction of sound duration and the corresponding increase in terminal frequency did not always reach a significant level if we compared the averages of the five calls before and after beginning of reaction (Fig. 2). These results reflect that some reactions were rather weak and short with only one or a few slightly shorter signals. This had a strong influence on the average value of the five signals after beginning of reaction so that the significance level was not reached with our five-call criterion. However, the differences between calls prior to reaction and after beginning of reaction were always significant in longer lasting reactions where bats produced more than five short reaction signals.

During encounters both bats often reacted with a distinct change in echolocation behavior (Figs 2,4 and 5). In pursuits, the pursued bat mostly reacted at the beginning of being pursued with a slight reduction of pulse duration, then returned to normal search behavior with longer signal durations (e.g. Bat 2 (blue) from t = 7.9–8.5 s, Fig. 3), whereas the pursuer emitted shorter signals for the entire time span of the pursuit (e.g. Bat 1 (red) from t = 7.8–10.0 s, Fig. 3).

Neither in encounters nor in pursuits we found any significant correlation between changes in echolocation behavior (reduction of call duration and pulse interval, increase of terminal frequency) and flight path angles to the conspecific (Spearman’s rank correlation, all p-values > 0.07 for sufficient sample sizes). For detailed information see Supplementary Tables S1 and S2.

During pursuits and encounters with signal durations below 3.5 ms, the terminal frequency was raised in a manner similar to approaches (Figs 1A,3C and 4C). In eight of 20 analyzed flight situations, residents reacting to an intruder also occasionally emitted one or a few short echolocation signals (call duration 0.5–3.5 ms) with distinctly higher terminal frequencies of up to 60 kHz (high frequency calls in Figs 3, 4, 5), which were interspersed between normal search calls. A total of 36 high frequency calls were recorded in 6 sequences, 12 of them during single flights and 24 during flights with a conspecific. A detailed description of these calls and a discussion of their communication function will be presented in a separate publication (Goetze, in prep).

To test whether *P. pipistrellus* uses the same signal repertoire when flying alone and with conspecifics, we investigated how well the transmission channels derived from single flight data predict the relation of duration and frequency of bats flying together and while interacting (C in Figs 3, 4, 5). From 847 search calls of all 20 analyzed flights with conspecifics, 89.7% (760 calls) were found within the predicted individual transmission channels.

## Discussion

The aim of this study was to understand the changes in echolocation behavior of foraging *Pipistrellus pipistrellus* while reacting to conspecifics during encounters and pursuits. The reaction to nearby conspecifics was indicated by distinct changes in the echolocation behavior of the involved individuals. Reaction distance between the reacting individual and the conspecific occurred across a wide range, between 1–11 m. In encounters, when bats flew towards one another, both bats frequently had distinct longer lasting reactions. In pursuits, only the pursuing bat typically showed such elongated reactions to the conspecific, while the pursued bat reacted only slightly or not at all. Sometimes bats with an inter-individual distance below 10 m did not react at all, even if they came as close as 3.5 m.

There have been two hypotheses to explain the observed changes in echolocation behavior of bats reacting to each other. The **jamming avoidance hypothesis** proposes that interacting individuals should separate the signal frequencies far enough such that spectral interference is reduced. In this case all reactions to conspecifics should always lead to a distinct separation of the terminal frequencies of the two bats, particularly when their individual frequencies are close together. We hypothesize-similar to the conclusion in the recent publication of Cvikel *et al.*^18^ - that the observed changes do not indicate JAR, but instead have the function of exploring the near-by conspecific which has captured the attention of the reacting bat. This **attention hypothesis** proposes that bats switch from search to attention behavior after being alerted to a conspecific near-by. The released attention behavior should be similar to other behavioral situations where targets of interest have captured the attention of bats and initiated a more detailed exploration. Such an attentional switch in echolocation behavior is known from bats flying in edge space and reacting to the background, characterized by a reduction of pulse duration and interval, and an increase of signal bandwidth according to the distance to the background (reviewed in^20^). Bats attending a target of interest such as prey, obstacles, or landing sites, switch to approach behavior which is also characterized by a distance-dependent change in echolocation parameters (reviewed in^19^). The reaction to background targets in edge space and to prey, obstacles, or landing sites during an approach is initiated by echo information received from these targets of interest.

We found that bats reacting to conspecifics changed their echolocation behavior in a similar way. Bats reduced sound duration and pulse interval and raised the terminal frequency according to predictions made by individual transmission channels. When comparing the five signals before a reaction with the following five signals the mean terminal frequency was significantly raised by 0.95 kHz which can be explained by a significant reduction of the mean sound duration by 1.27 ms. The observed increase in terminal frequency can be explained by the attention hypothesis alone.

In 26.7% of analyzed flights with conspecifics the frequency difference between bats did increase, which could be interpreted as evidence for spectral JAR. However, in each of these cases the bat with the higher individual frequency reacted either alone or with a stronger reduction of sound duration than the conspecific, which equally accounts for the observed increase. In addition, many other cases were observed where the bat with the lower terminal frequency reacted alone or where the frequency difference between the two bats diminished, evidence that contradicts a JAR hypothesis. In bats with similar reaction amplitudes, the frequency was shifted upward in parallel. Another argument against the JAR hypothesis is that bats shifted their terminal frequency upwards independently from the absolute frequency difference between the reacting individuals. In summary, our data support the attention hypothesis and reject the JAR hypothesis.

But why do bats direct their attention to a conspecific flying near-by? Cvikel *et al.*^18^ came to the conclusion that *Rhinopoma microphyllum* attend a conspecific similar to an obstacle which has entered their acoustic field of view and react to it in a similar manner, with a shortening of the echolocation signals accompanied by a rise in frequency. Under this hypothesis, bats use echoes from their own echolocation signals for an active perception of the conspecific. This implies that the conspecific must be within the detection distance of the bat. Our data show that *P. pipistrellus* reacted to conspecifics up to a distance of 11 m. Assuming for *P. pipistrellus* in search flight a terminal frequency of 45 kHz, an emission SL of 106 dB (re 1 m), a detection threshold of 20 dB, a target strength of −40 dB^40^ for the conspecific, a temperature of 20 °C, and humidity of 50%, we calculated a maximum detection distance of 5.4 m for the active perception of the other bat by echolocation, but only if the other bat was straight ahead^41^. Besides detection, localization, and identification by active echolocation on a rather short distance, bats can also perceive each other over a much wider distance by passive acoustic localization of continuously emitted echolocation signals. Our calculations revealed a maximum detection distance of ~37 m for the passive perception of signals from pipistrelle bats. Therefore, we broaden the hypothesis of Cvikel *et al.*^18^ and assume that bats react to conspecifics not only on the basis of information gained by echolocation, but also from passive acoustic information to control the behavior of conspecifics. Information on position, movement pattern, and behavioral status a bat gains either by passive or active perception provides a basis for the decision whether a conspecific needs more exploration. If additional attention is required, the bat can then switch to attention behavior which delivers more detailed information via active echolocation.

Our results, as well as those of Cvikel *et al.*^18^, do not support a spectral JAR hypothesis for either *P. pipistrellus* nor *R. microphyllum*, which is most likely applicable to other bat species. This contradicts many publications which postulate that bats use JAR to prevent interferences produced by the echolocation signals and echoes from other bats^1^,^2^,^3^,^4^,^5^,^6^,^7^,^8^,^9^,^10^,^11^,^12^. It is possible that changes in the search behavior of bats, e.g. the use of different types of search signals, have been mistakenly identified as a spectral JAR. To overcome this uncertainty, we suggest several lines of evidence that should be observed prior to a response being identified as a JAR; 1) the spatial relationship between the interacting bats should be known and the inter-individual distance should be small enough that an interaction is reasonable, 2) the interaction should lead to an increased frequency difference between the echolocation signals of the interacting bats, and 3) these differences in frequency should be consistent and different enough such that they cannot solely be explained by a switch to attention behavior.

The result of our field data that foraging bats react to conspecifics with attention behavior and not with a JAR does not exclude that jamming signals evoke reactions in bats which could be interpreted as JAR. For instance, in horseshoe bats (*Rhinolophus ferrumequinum*), strong bandpass-filtered noise affected call amplitude and frequency which was explained by the Lombard effect^42^. This effect describes an involuntary rise in call amplitude in response to masking noise and optimizes signal-to-noise-ratios. Luo *et al.*^43^ found an increase in SPL, duration, and repetition rate in signals of *Phyllostomus discolor* as a response to increasing noise levels. These changes, which improved signal detectability, were also attributed to the Lombard effect. This effect may also explain why big brown bats (*Eptesicus fuscus)* reacted to increasing noise levels in a range discrimination task with an increase in sound duration and SPL^44^. In a recent study, Amichai *et al.*^37^ compared reactions of *Pipistrellus kuhlii* to very loud signals of conspecifics while flying and landing in a flight room. The bats responded with reactions predicted by the Lombard effect, and emitted calls with higher SPLs and longer durations at higher repetition rates. Small spectral shifts were also observed but no increase in frequency differences between the calls was observed. The authors concluded that the observed responses were made to increase the signal-to-noise-ratio and not to avoid spectral overlap.

From all publications reporting spectral jamming avoidance, only the study of Bates *et al.*^13^ has strong evidence for this behavior. In this study, stationary *E. fuscus* were confronted with a constant frequency jamming tone while performing a two-alternative forced-choice detection task. The target echoes and the jamming tone had approximately the same sensation level of 65 dB. Bats shifted the terminal frequency of their signals away from the jamming signal as long as the jamming frequency was closer than 2–3 kHz to their preferred frequency, but they did not change signal duration. They concluded that this jamming avoidance response may serve to avoid masking or interference during target detection. These data may indicate that stationary bats exposed to a continuous tone of a sufficient SPL move their frequency away from the jamming signal but only within a narrow range. This small range is explained by our findings that the width of individual transmission channels is limited.

Another aim of this study was to test whether the correlation between sound duration and terminal frequency is suited for the characterization of the signal repertoire of individual *P. pipistrellus* and also of the species. For the quantification of this correlation we used a method developed by Baumann^34^ and determined the transmission channels for search signals of individual bats when flying alone, and for the species, from all recorded search signals in single flight. We found a negative correlation between terminal frequency and signal duration, consistent with other studies of pipistrelle and other vespertilionid bats^3^,^25^,^27^,^30^,^31^,^32^,^33^,^34^,^35^. The species-specific transmission channel calculated from the double standard deviation of the residuals had a width of 4.5 kHz, whereas the average width of the individual channels was only 2.7 kHz. Individual terminal frequencies as indicated by the average frequencies of calls with durations of 5–6 ms of single flying *P. pipistrellus* varied over a wide range, from 43.5 kHz to 49.1 kHz. With this approach we showed for the first time that the transmission channels of the terminal frequencies of individual bats are distinctly smaller than the transmission channel of the species. We also found that the signal repertoires of individuals flying together are similar to the repertoires used in single flight. The frequencies of nearly all signals (89.7% from 847 calls) from interacting bats were within the predicted individual transmission channels.

But why don’t foraging pipistrelle bats need to shift their terminal frequencies apart to prevent potential jamming? Jamming could occur if a bat’s echoes were masked by loud calls of a conspecific or if a bat mistook the call of a conspecific (and/or its echoes) for its own and evaluated these external echoes together with its own pulse as a coherent pulse-echo-train. The latter is rather unlikely. The individual terminal frequencies of pipistrelle bats spanned a wide range from 43.5–49.1 kHz. Additionally, the duration of search signals varied. An accidental fit in terminal frequency and duration between the emitted signal of a bat and the jamming signals from a conspecific would therefore be very rare. However, even if such coincidences could occur, jamming is still unlikely. Bats can use echolocation signals to recognize voices based on echolocation calls^38^ and should therefore be able to discriminate their own voice encoded in the echoes from the echoed signals of conspecifics. Additionally, bats only evaluate reasonable pulse-echo relations such that the perceived auditory scenes make sense. Irrelevant echoes which do not fit into this criteria will be ignored^45^. Furthermore, evidence suggests that neuronal gating mechanisms prevent irrelevant echoes that do not return in a proper time window after signal emission from being processed^46^. Finally, bats can also use the high directionality of their sound emission and hearing system to suppress unwanted interferences^47^. All of these mechanisms together make the echolocation systems of bats unlikely to be interfered under foraging conditions, so that no additional jamming avoidance mechanisms need be implemented. It could be argued that under particularly extreme conditions (e.g. if hundreds of bats passing the narrow entrance of a cave) myriad loud calls of conspecifics could potentially cause severe jamming. In such a situation, however, it is unlikely that spectral JAR would help, as all frequency channels would already be in use. In this case, additional mechanisms such as the emission of louder signals with an increased signal-to-noise-ratio^37^, and/or the use of spatial memory and vision could be utilized.

## Conclusion

When flying alone or together, pipistrelle bats use the same signal repertoire described by the individual transmission channels that are based on the correlation between sound duration and terminal frequency. In foraging *P. pipistrellus*, a nearby flying conspecific may capture a bat’s attention either by passive acoustic localization and evaluation of the echolocation signals over large distances, or by active echolocation at short distances. If spatial position, movement pattern, and behavioral situation of the conspecific are relevant to the bat, it will explore the subject with echolocation in more detail and switch to attention behavior with shorter signals, higher terminal frequency, and reduced pulse intervals. All observed changes of terminal frequency in our study reflect this reaction pattern and do not support the spectral JAR hypothesis.

## Additional Information

**How to cite this article**: Götze, S. *et al.* No evidence for spectral jamming avoidance in echolocation behavior of foraging pipistrelle bats. *Sci. Rep.* **6**, 30978; doi: 10.1038/srep30978 (2016).

## Supplementary Material

Supplementary Information

## Figures and Tables

**Table 1:** Reactions of pipistrelle bats during pursuits.

	Δ individual terminal frequency by cd 5.5 ms [kHz]	Sequence	Pursuer	Individual	Individual terminal frequency by cd 5.5.ms (mean ± sd) [kHz]	Reaction distance [m]	Minimal inter-individual distance [m]	n calls in flight with conspecific	n calls in flight with conspecific by cd 3.5–9.5 ms	n in transmission channel in flight with conspecific by cd 3.5–9.5 ms	[%] in transmission channel in flight with conspecific by cd 3.5–9.5 ms	Attention hypothesis	JAR hypothesis
**pursuits**	<1	3b	P	Bat 1 Bat 2*	47.0 ± 1.0 46.5 ± 0.7	6.0 6.7	1.2	38 46	38 27	38 23	100 85.2	+	
		2a	P	Bat 1* Bat 2	47.5 ± 0.6 47.6	5.0 6.6	1.1	24 19	16 19	15 19	93.8 100	+	+
	2–3	12b	P	Bat 1* Bat 2	46.6 ± 0.5 48.8 ± 0.5	5.3 5.2	4.3	15 17	3 17	2 16	66.7 94.1	+	
		1b	P	Bat 1* Bat 2	46.6 ± 0.7 48.8 ± 0.6	0.7 −	0.6	24 13	17 13	14 13	82.4 100	+	
		10	P	Bat 1 Bat 2	44.9 ± 0.8 47.1 ± 0.8	9.0 6.7	4.3	41 39	35 37	33 27	94.3 73.0	+	+
		15	P	Bat 1 Bat 2	44.9 ± 0.8 47.1 ± 0.8	1.6 3.7	1.5	39 48	38 20	36 9	94.7 45.0	+	+
	3–4	17	P	Bat 1 Bat 2	48.2 ± 0.8 45.1 ± 0.5	− −	3.5	33 43	33 36	25 36	75.8 100	+	+
		14	P	Bat 1* Bat 2	49.1 ± 0.4 45.1 ± 0.5	2.6 1.2	0.6	48 44	21 44	21 42	100 95.5	+	
		8		Bat 1 Bat 2	47.1 ± 0.2 43.2 ± 0.8	− −	3.6	38 38	38 29	30 28	78.9 96.6	+	

Residents are marked with asterisks (*). Reaction distances without values (−) mark non-reacting individuals, cd = call durations.

**Table 2:** Reactions of pipistrelle bats during encounters.

	Δ individual terminal frequency by cd 5.5 ms [kHz]	Sequence	Individual	Individual terminal frequency by cd 5.5. ms (mean ± sd) [kHz]	Reaction distance [m]	Minimal inter-individual distance [m]	n calls in flight with conspecific	n calls in flight with conspecific by cd 3.5–9.5 ms	n in transmission channel in flight with conspecific by cd 3.5–9.5 ms	[%] in transmission channel in flight with conspecific by cd 3.5–9.5 ms	Attention hypothesis	JAR hypothesis
**encounter**	<1	3a	Bat 1 Bat 2	47.0 ± 1.0 46.5 ± 0.7	2.8 5.4	1.2	21 26	21 21	20 19	95.2 90.5	+	
		7	Bat 1 Bat 2*	47.1 ± 0.6 47.4 ± 0.7	10.8 3.5	3.5	24 27	24 27	20 26	83.3 96.3	+	
		2b	Bat 1* Bat 2	47.5 ± 0.6 47.6	3.2 3.9	1.1	18 19	14 17	11 17	78.6 100	+	+
	1–2	4a	Bat 1* Bat 2	45.0 ± 0.6 46.5	6.2 7.8	1.9	21 19	19 18	19 12	100 66.6	+	+
		6	Bat 1* Bat 2	46.9 ± 0.5 45.5 ± 0.5	4.9 −	2.8	19 19	16 19	16 19	100 100	+	
	2–3	12a	Bat 1* Bat 2	46.6 ± 0.5 48.8 ± 0.5	8.1 8.9	4.3	8 10	8 10	7 10	87.5 100	+	
		1a	Bat 1* Bat 2	46.6 ± 0.7 48.8 ± 0.6	1.5 1.5	0.6	18 15	12 15	11 15	91.7 100	+	
		9	Bat 1 Bat 2	44.9 ± 0.8 47.1 ± 0.8	5.0 6.8	3.3	31 17	31 16	27 12	87.1 75	+	
		11	Bat 1* Bat 2	44.9 ± 0.8 47.1 ± 0.8	10.3 4.5	4.3	15 9	15 9	15 8	100 88.9	+	
		5	Bat 1* Bat 2	43.5 ± 0.8 46.2 ± 0.7	X 3.2	2.8	14 11	9 11	8 11	88.9 100	+	
	3–4	13	Bat 1 Bat 2	48.2 ± 0.8 45.1 ± 0.5	− −	7.4	15 19	15 19	12 18	80 94.7	+	+

Reaction distances without values (−) mark non-reacting individuals, cd = call duration. Reaction distance value marked by ‘X’ could. not be measured.
