# Supplementary material for: No evidence for spectral jamming avoidance in echolocation behavior of foraging pipistrelle bats
Source: Sci Rep. 2016 Aug 9;6:30978. doi: 10.1038/srep30978 (PMC4977515; doi:10.1038/srep30978)
Supplement: Supplementary Information [file srep30978-s1.pdf]

No evidence for spectral jamming avoidance in echolocation behavior of foraging pipistrelle bats

Simone Götze[1]*, Jens C. Koblitz[2], Annette Denzinger[1], Hans-Ulrich Schnitzler[1]

[1] University of Tuebingen, Department Animal Physiology, Institute for Neurobiology, Auf der Morgenstelle 28, 72076 Tuebingen, Germany

[2] BioAcoustics Network, Neuss, Germany

Correspondence and requests for materials should be addressed to S.G. (email: simone.goetze@student.uni-tuebingen.de)

Supplemental Material

Table S1: Spearman's rank correlation between changes in echolocation behavior and flight path angles to the conspecific during encounters.

Encounters	Angle to conspecific		
	ρ (Rho)	p	N
Terminal frequency shift	-0.1912	0.4329	19
Call duration reduction	-0.2719	0.2601	19
Pulse interval reduction	-0.2825	0.2413	19

Table S2: Spearman's rank correlation between changes in echolocation behavior and flight path angles to the conspecific during pursuits.

	Pursuer			Pursued Bat		
Pursuits	Angle to conspecific			Angle to conspecific		
	ρ (Rho)	p	N	ρ (Rho)	p	N
Terminal frequency shift	-0.2	0.704	6	-0.7818	0.0378	7
Call duration reduction	0.3143	0.5441	6	-0.3091	0.5	7
Pulse interval reduction	-0.7714	0.0724	6	-0.1441	0.7578	7

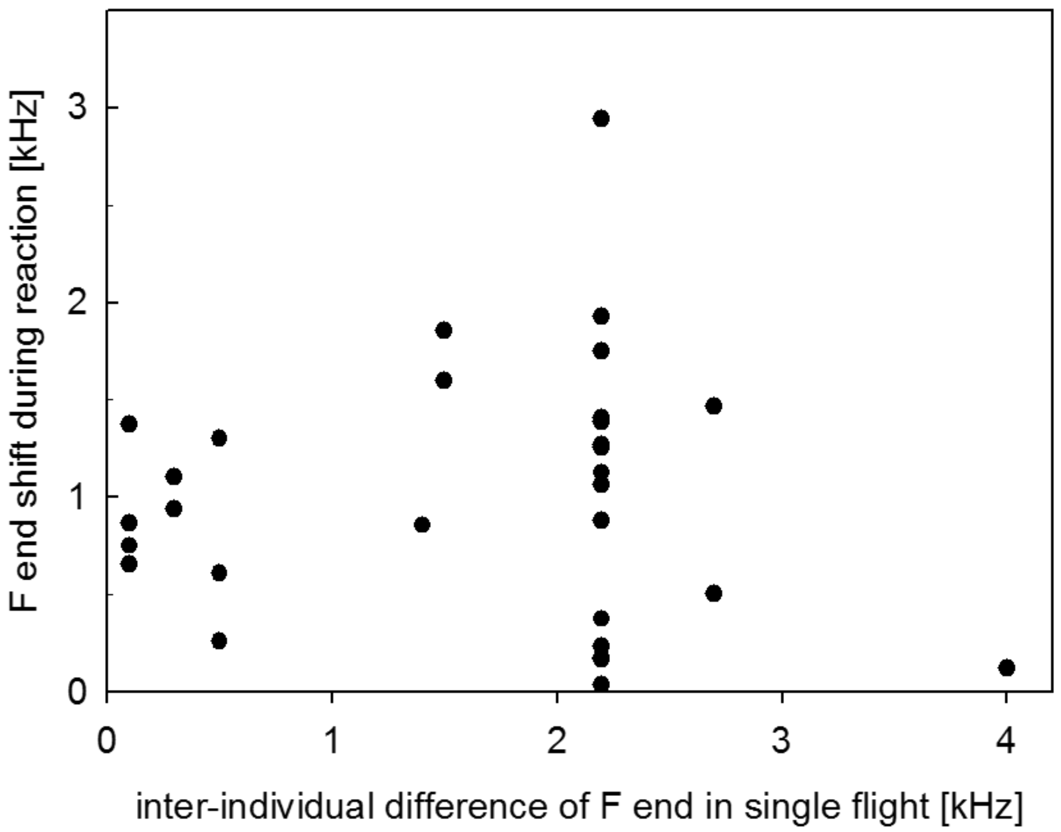

Supplemental Fig. S1

Upward shifts of terminal frequency in reacting bats at absolute frequency differences between the interacting individuals.

The observed increase of terminal frequency during reaction to a conspecific is not correlated with the absolute inter-individual frequency difference between the interacting bats (Spearman’s ρ, R = -0.03, N=31, p=0.88). Individual frequencies have been determined from single flight calls with durations of 5-6 ms. F end = Terminal frequency
